# Crystal structure of 5′′-(4-chloro­benzyl­idene)-4′-(4-chloro­phen­yl)-1′-methyltri­spiro[acenapthylene-1,2′-pyrrolidine-3′,1′′-cyclo­hexane-3′′,2′′′-[1,3]dioxane]-2(1*H*),6′′-dione

**DOI:** 10.1107/S2056989015018034

**Published:** 2015-10-03

**Authors:** Kuppan Chandralekha, Deivasigamani Gavaskar, Adukamparai Rajukrishnan Sureshbabu, Srinivasakannan Lakshmi

**Affiliations:** a Research Department of Physics, S. D. N. B. Vaishnav College for Women, Chromepet, Chennai 600 044, India; b Department of Organic Chemistry, University of Madras, Guindy Campus, Chennai 600 025, India

**Keywords:** crystal structure, spiro pyrrolidines, ace­naphthyl­ene, dioxalane, hydrogen bonding

## Abstract

In the title compound, C_36_H_29_Cl_2_NO_4_, two spiro links connect the methyl-substituted pyrrolidine ring to the ace­naphthyl­ene and cyclo­hexa­none rings. The cyclo­hexa­none ring is further connected to the dioxalane ring by a third spiro junction. The five-membered ring of the ace­naphthylen-1-one ring system adopts a flattened envelope conformation, with the ketonic C atom as the flap, whereas the dioxalane and pyrrolidine rings each have a twist conformation. The cyclo­hexenone ring assumes a boat conformation. An intra­molecular C—H⋯O hydrogen-bond inter­action is present. In the crystal, mol­ecules are linked by non-classical C—H⋯O hydrogen bonds, forming chains extending parallel to the *a* axis.

## Related literature   

For the pharmacological properties of spiro compounds, see: Cravotto *et al.* (2001 ▸); Raj *et al.* (2003 ▸); Stylianakis *et al.* (2003 ▸). For the activities of ace­naphthyl­ene derivatives, see: Selvanayagam *et al.* (2004 ▸); El-Ayaan *et al.* (2007 ▸); McDavid & Daniels (1951 ▸); El-Ayaan & Abdel-Aziz (2005 ▸); Smith *et al.* (1979 ▸); Chen *et al.* (2014 ▸). For the properties and pharmacological activities of dioxalane compounds, see: Narayanasamy *et al.* (2007 ▸); Küçük *et al.* (2011 ▸); Shirai *et al.* (1998 ▸); Bera *et al.* (2003 ▸); Aepkers & Wünsch (2005 ▸); Ozkanlı *et al.* (2003 ▸); Liang *et al.* (2006 ▸).
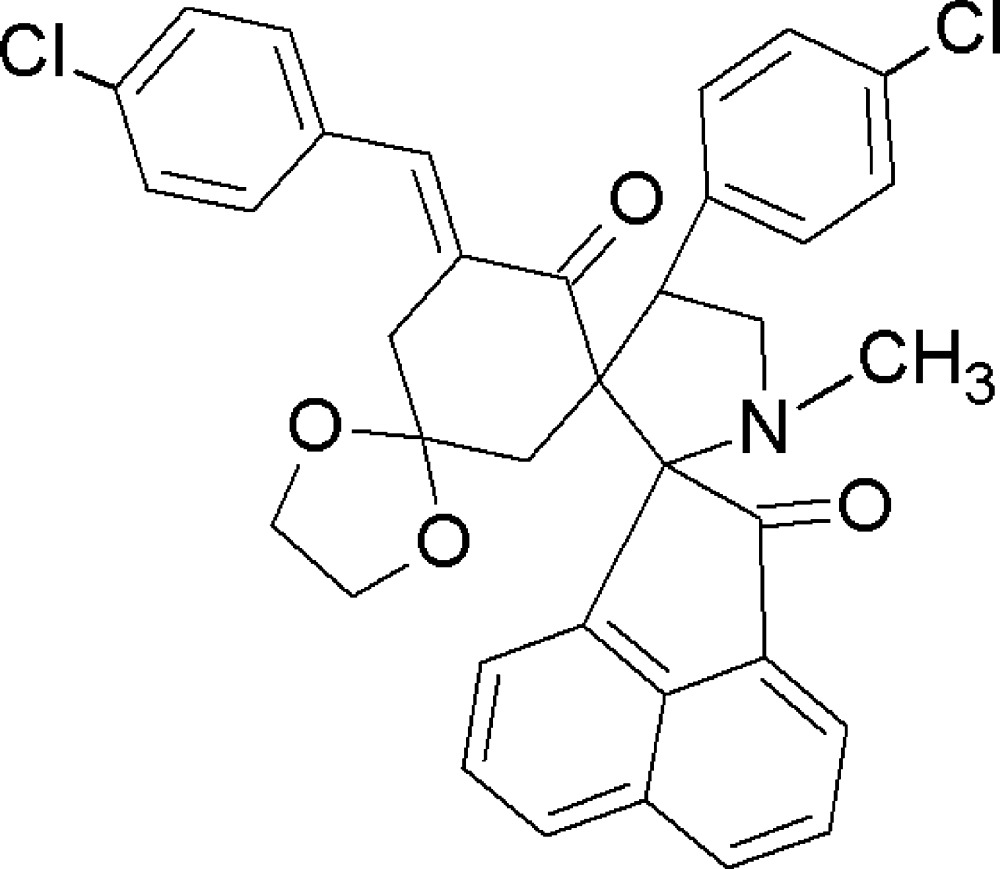


## Experimental   

### Crystal data  

C_36_H_29_Cl_2_NO_4_
*M* *_r_* = 610.50
Triclinic 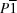

*a* = 8.9791 (4) Å
*b* = 10.3080 (5) Å
*c* = 15.7653 (6) Å
α = 88.679 (2)°
β = 83.263 (2)°
γ = 87.408 (2)°
*V* = 1447.39 (11) Å^3^
*Z* = 2
Mo *K*α radiation
μ = 0.27 mm^−1^
*T* = 293 K
0.35 × 0.30 × 0.25 mm

### Data collection  

Bruker Kappa APEXII CCD diffractometer
Absorption correction: multi-scan (*SADABS*; Bruker, 2004 ▸) *T*_min_ = 0.708, *T*_max_ = 0.746
39174 measured reflections
5104 independent reflections
3981 reflections with *I* > 2σ(*I*)
*R*_int_ = 0.027

### Refinement  

*R*[*F* ^2^ > 2σ(*F* ^2^)] = 0.041
*wR*(*F* ^2^) = 0.111
*S* = 1.06
5104 reflections
389 parameters
H-atom parameters constrained
Δρ_max_ = 0.47 e Å^−3^
Δρ_min_ = −0.33 e Å^−3^

### 

Data collection: *APEX2* (Bruker, 2004 ▸); cell refinement: *SAINT* (Bruker, 2004 ▸); data reduction: *SAINT*; program(s) used to solve structure: *SHELXS97* (Sheldrick, 2008 ▸); program(s) used to refine structure: *SHELXL2014* (Sheldrick, 2015 ▸); molecular graphics: *ORTEP-3 for Windows* (Farrugia, 2012 ▸) and *PLATON* (Spek,2009 ▸); software used to prepare material for publication: *publCIF* (Westrip, 2010 ▸).

## Supplementary Material

Crystal structure: contains datablock(s) I. DOI: 10.1107/S2056989015018034/rz5168sup1.cif

Structure factors: contains datablock(s) I. DOI: 10.1107/S2056989015018034/rz5168Isup2.hkl

Supporting information file. DOI: 10.1107/S2056989015018034/rz5168Isup3.cml

**Fig. 1.**
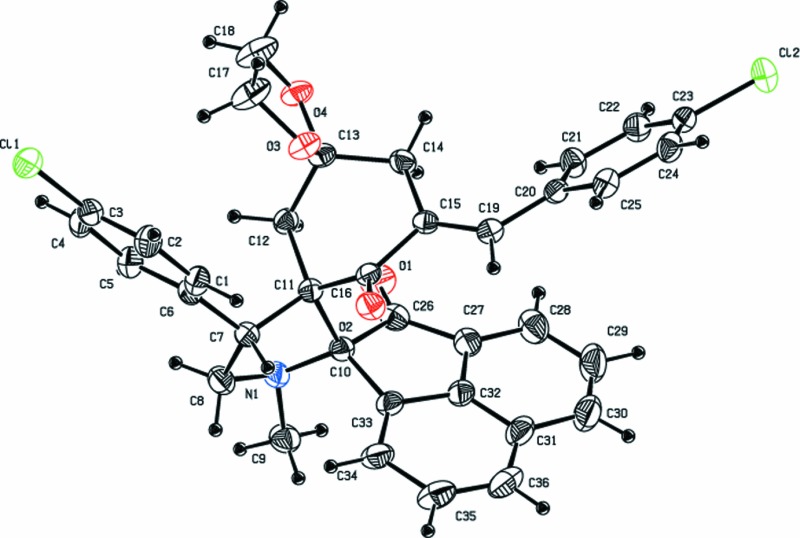
The molecular structure of the title compound, with displacement ellipsoids drawn at the 30% probability level. H atoms are shown as small spheres of arbitrary radius.

**Fig. 2.**
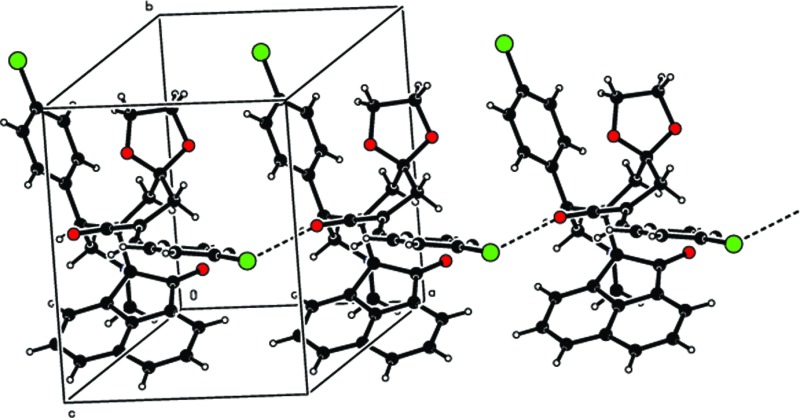
Partial crystal packing of the title compound showing the formation of a molecular chain parallel to the a axis via C—H···O hydrogen bonds (dashed lines).

. DOI: 10.1107/S2056989015018034/rz5168fig1.tif
The mol­ecular structure of the title compound, with displacement ellipsoids drawn at the 30% probability level. H atoms are shown as small spheres of arbitary radius.

a via . DOI: 10.1107/S2056989015018034/rz5168fig2.tif
Partial crystal packing of the title compound showing the formation of a mol­ecular chain parallel to the *a* axis *via* C—H⋯O hydrogen bonds (dashed lines).

CCDC reference: 1427830

Additional supporting information:  crystallographic information; 3D view; checkCIF report

## Figures and Tables

**Table 1:** Hydrogen-bond geometry (, )

*D*H*A*	*D*H	H*A*	*D* *A*	*D*H*A*
C12H12*A*O1	0.97	2.27	3.066 (3)	139
C22H24O2^i^	0.93	2.35	3.172 (3)	148

Symmetry code: (i) 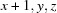.

## supplementary crystallographic information

### S1. Comment

Spiro compounds frequently form a part of pharmacologically relevant alkaloids (Cravatto *et al.*, 2001). Spiro pyrrolidines are an important class of compounds having anti­bacterial and anti­fungal activities against human pathogenic bacteria and dermatophytic fungi (Amal Raj *et al.*, 2003), and are active against anti-influenza virus A. (Styliankis *et al.*, 2003). Ace­naphthyl­ene derivatives are found to have high κ-opiod receptor affinity and selectivity (Selvanayagam *et al.*, 2004). These derivatives have anti­tumor (Ayaan *et al.*, 2007), anti­fungal (McDavid & Daniels, 1951), anti­microbial (Ayaan & Abdel-Aziz, 2005), anti-inflammatory (Smith *et al.*, 1979) and insecticidal activities (Chen *et al.*, 2014). Dioxalane compounds exihibit anti-HIV (Narayanasamy *et al.*, 2007), anti­bacterial and anti­fungal (Kucuk *et al.*, 2011), anti­neoplastic (Shirai *et al.*, 1998), anti­viral (Bera *et al.*, 2003), anaesthetic (Aepkers & Wünsch, 2005) and anti­convulsant activities (Ozkanlı *et al.*, 2003). Dioxalane moieties play also a significant role in stabilizing the binding between the mutant HIV-1 RT and nucleoside triphosphate and act as nucleoside reverse transcriptase inhibitors (NRTIs) (Liang *et al.*, 2006).

In the title compound (Fig. 1), the methyl substituted pyrrolidine ring (C7/C8/N/C9/C10/C11), is in twist conformation with puckering parameters q2 = 0.454 (2) Å, φ = 127.8 (3)° .The dioxalane ring (C13/O3/C17/C18/O4) has also a twist conformation (q2 = 0.202 (3) Å, φ = -127.6 (7)°), while the five-membered ring (C10/C26/C27/C32/C33) of the ace­naphthylen-1-one ring system adopts a flattened envelope conformation (q2 = 0.112 (2) Å, φ = 26.8 (11)°). The six-membered cyclo­hexanone ring (C11—C16) adopts a boat conformation (Q_T_ = 0.690 (2) Å, Θ = 99.72 (16)°, φ = 9.84 (16)°). The least-squares mean plane through the pyrrolidine ring forms dihedral angles of 120 (18), 90.55 (7) and 97.57 (8)° with the mean planes of the attached benzene ring, cyclo­hexanone ring and cyclo­penta­none ring, respectively. The mean planes through the cyclo­hexanone and dioxalane rings form a dihedral angle of 92.61 (10)°. The sum of bond angles around the nitro­gen atom of the pyrrolidine ring (338.4°) is in agreement with an *sp*^3^ hybridization. The molecular conformation is stabilized by an intra­molecular C—H···O hydrogen bond (Table 1). In the crystal (Fig. 2), molecules are linked by weak inter­molecular C—H···O hydrogen inter­actions (Table 1) to form chains extending parallel to the *a* axis.

### S2. Experimental

An equimolar mixture of 7,9-bis­[(*E*)-aryl­idene-1,4-dioxo-spiro­[4,5]decane-8-one (1 mmol), acenapthe­quinone (1 mmol) and sarcosine in methanol (25-30 ml) was refluxed for 4 hours. After completion of the reaction as indicated by TLC, the solid precipitate was filtered and washed with methanol to give the pure tri­spiro­pyrrolidine derivative. Single crystals suitable for the X-ray diffraction analysis were obtained by slow evaporation of the solvent at room temperature.

### S3. Refinement

All H atoms were placed in calculated positions, with C—H = 0.93–0.98 Å and refined using a riding-model approximation, with *U*_iso_(H) = 1.2 *U*_eq_(C) or 1.5 *U*_eq_(C) for methyl H atoms. A rotating model was applied to the methyl groups.

### Crystal data

**Table:**

C_36_H_29_Cl_2_NO_4_	*V* = 1447.39 (11) Å^3^
*M**_r_* = 610.50	*Z* = 2
Triclinic, *P*1	*F*(000) = 636
Hall symbol: -P 1	*D*_x_ = 1.401 Mg m^−^^3^
*a* = 8.9791 (4) Å	Mo *K*α radiation, λ = 0.71073 Å
*b* = 10.3080 (5) Å	θ = 1.3–25.0°
*c* = 15.7653 (6) Å	µ = 0.27 mm^−^^1^
α = 88.679 (2)°	*T* = 293 K
β = 83.263 (2)°	Block, colourless
γ = 87.408 (2)°	0.35 × 0.30 × 0.25 mm

### Data collection

**Table:**

Bruker Kappa APEXII CCD diffractometer	5104 independent reflections
Radiation source: fine-focus sealed tube	3981 reflections with *I* > 2σ(*I*)
Graphite monochromator	*R*_int_ = 0.027
bruker axs kappa apex2 CCD Diffractometer scans	θ_max_ = 25.0°, θ_min_ = 2.0°
Absorption correction: multi-scan (*SADABS*; Bruker, 2004)	*h* = −10→10
*T*_min_ = 0.708, *T*_max_ = 0.746	*k* = −12→12
39174 measured reflections	*l* = −18→18

### Refinement

**Table:**

Refinement on *F*^2^	0 restraints
Least-squares matrix: full	Hydrogen site location: inferred from neighbouring sites
*R*[*F*^2^ > 2σ(*F*^2^)] = 0.041	H-atom parameters constrained
*wR*(*F*^2^) = 0.111	*w* = 1/[σ^2^(*F*_o_^2^) + (0.0381*P*)^2^ + 1.0322*P*] where *P* = (*F*_o_^2^ + 2*F*_c_^2^)/3
*S* = 1.06	(Δ/σ)_max_ < 0.001
5104 reflections	Δρ_max_ = 0.47 e Å^−^^3^
389 parameters	Δρ_min_ = −0.33 e Å^−^^3^

### Special details

**Table:**

Geometry. All e.s.d.'s (except the e.s.d. in the dihedral angle between two l.s. planes) are estimated using the full covariance matrix. The cell e.s.d.'s are taken into account individually in the estimation of e.s.d.'s in distances, angles and torsion angles; correlations between e.s.d.'s in cell parameters are only used when they are defined by crystal symmetry. An approximate (isotropic) treatment of cell e.s.d.'s is used for estimating e.s.d.'s involving l.s. planes.

### Fractional atomic coordinates and isotropic or equivalent isotropic displacement parameters (Å^2^)

**Table:**

	*x*	*y*	*z*	*U*_iso_*/*U*_eq_	
Cl1	0.45365 (8)	0.89061 (6)	0.09442 (5)	0.0637 (2)	
Cl2	1.57635 (8)	0.32960 (7)	0.57298 (5)	0.0672 (2)	
O1	1.1573 (2)	0.16684 (18)	0.11243 (12)	0.0626 (5)	
O2	0.75080 (16)	0.36814 (16)	0.33228 (9)	0.0459 (4)	
O3	0.93988 (18)	0.60388 (15)	0.22420 (10)	0.0488 (4)	
O4	1.14319 (18)	0.59140 (16)	0.12382 (10)	0.0513 (4)	
N1	0.8362 (2)	0.16901 (18)	0.09369 (12)	0.0424 (4)	
C3	0.5243 (3)	0.7341 (2)	0.11282 (16)	0.0455 (6)	
C4	0.5993 (3)	0.6655 (2)	0.04656 (15)	0.0503 (6)	
H2	0.6140	0.7031	−0.0078	0.060*	
C5	0.6531 (3)	0.5403 (2)	0.06081 (15)	0.0473 (6)	
H3	0.7022	0.4934	0.0154	0.057*	
C2	0.5025 (3)	0.6802 (2)	0.19309 (16)	0.0507 (6)	
H4	0.4508	0.7269	0.2379	0.061*	
C1	0.5579 (3)	0.5557 (2)	0.20679 (15)	0.0454 (5)	
H5	0.5431	0.5192	0.2615	0.055*	
C6	0.6351 (2)	0.4832 (2)	0.14162 (14)	0.0400 (5)	
C7	0.6956 (2)	0.3479 (2)	0.16184 (14)	0.0393 (5)	
H7	0.6299	0.3155	0.2111	0.047*	
C8	0.6974 (3)	0.2474 (2)	0.09179 (16)	0.0508 (6)	
H8A	0.6112	0.1935	0.1027	0.061*	
H8B	0.6950	0.2901	0.0365	0.061*	
C9	0.8264 (3)	0.0346 (2)	0.06982 (17)	0.0550 (6)	
H9A	0.9222	−0.0101	0.0717	0.082*	
H9B	0.7983	0.0318	0.0130	0.082*	
H9C	0.7524	−0.0066	0.1090	0.082*	
C10	0.8921 (2)	0.1864 (2)	0.17486 (13)	0.0375 (5)	
C11	0.8580 (2)	0.3381 (2)	0.18748 (13)	0.0353 (5)	
C12	0.9679 (2)	0.4220 (2)	0.12925 (13)	0.0384 (5)	
H12A	1.0427	0.3656	0.0973	0.046*	
H12B	0.9131	0.4705	0.0886	0.046*	
C13	1.0458 (2)	0.5155 (2)	0.17975 (14)	0.0396 (5)	
C17	0.9409 (4)	0.7206 (3)	0.1776 (2)	0.0748 (9)	
H14A	0.9291	0.7940	0.2156	0.090*	
H14B	0.8603	0.7256	0.1416	0.090*	
C18	1.0865 (4)	0.7201 (3)	0.1258 (2)	0.0809 (10)	
H15A	1.0755	0.7521	0.0685	0.097*	
H15B	1.1539	0.7751	0.1508	0.097*	
C14	1.1287 (2)	0.4436 (2)	0.24586 (14)	0.0413 (5)	
H16A	1.1970	0.3772	0.2188	0.050*	
H16B	1.1867	0.5033	0.2739	0.050*	
C15	1.0139 (2)	0.3824 (2)	0.31022 (13)	0.0348 (5)	
C16	0.8631 (2)	0.36672 (19)	0.28166 (13)	0.0350 (5)	
C23	1.4171 (3)	0.3371 (2)	0.51992 (15)	0.0436 (5)	
C20	1.1664 (2)	0.3461 (2)	0.43347 (13)	0.0367 (5)	
C25	1.1547 (3)	0.3680 (2)	0.52061 (14)	0.0433 (5)	
H21	1.0604	0.3854	0.5503	0.052*	
C24	1.2791 (3)	0.3647 (2)	0.56416 (14)	0.0470 (6)	
H22	1.2697	0.3807	0.6224	0.056*	
C19	1.0309 (2)	0.3480 (2)	0.39060 (13)	0.0375 (5)	
H23	0.9447	0.3217	0.4239	0.045*	
C22	1.4334 (3)	0.3129 (2)	0.43433 (15)	0.0470 (6)	
H24	1.5278	0.2929	0.4055	0.056*	
C21	1.3083 (3)	0.3183 (2)	0.39151 (14)	0.0447 (5)	
H25	1.3192	0.3031	0.3332	0.054*	
C26	1.0622 (3)	0.1453 (2)	0.17153 (15)	0.0446 (5)	
C27	1.0841 (3)	0.0720 (2)	0.25048 (16)	0.0470 (6)	
C28	1.2105 (3)	0.0226 (3)	0.2827 (2)	0.0642 (7)	
H28	1.3058	0.0357	0.2547	0.077*	
C29	1.1902 (4)	−0.0486 (3)	0.3599 (2)	0.0792 (10)	
H29	1.2747	−0.0815	0.3834	0.095*	
C30	1.0535 (4)	−0.0715 (3)	0.4015 (2)	0.0762 (9)	
H30	1.0464	−0.1198	0.4523	0.091*	
C31	0.9216 (3)	−0.0235 (2)	0.36937 (17)	0.0576 (7)	
C32	0.9431 (3)	0.0500 (2)	0.29316 (15)	0.0453 (6)	
C33	0.8251 (3)	0.1058 (2)	0.25144 (14)	0.0421 (5)	
C34	0.6825 (3)	0.0812 (2)	0.28446 (18)	0.0566 (7)	
H34	0.6013	0.1125	0.2573	0.068*	
C35	0.6596 (4)	0.0064 (3)	0.3614 (2)	0.0721 (8)	
H35	0.5617	−0.0099	0.3842	0.086*	
C36	0.7742 (4)	−0.0422 (3)	0.40321 (19)	0.0705 (8)	
H36	0.7540	−0.0881	0.4545	0.085*	

### Atomic displacement parameters (Å^2^)

**Table:**

	*U*^11^	*U*^22^	*U*^33^	*U*^12^	*U*^13^	*U*^23^
Cl1	0.0657 (4)	0.0445 (4)	0.0836 (5)	0.0004 (3)	−0.0236 (4)	0.0104 (3)
Cl2	0.0661 (4)	0.0741 (5)	0.0669 (4)	−0.0023 (4)	−0.0323 (3)	0.0069 (3)
O1	0.0480 (10)	0.0648 (12)	0.0705 (12)	0.0030 (9)	0.0100 (9)	−0.0047 (9)
O2	0.0358 (9)	0.0582 (10)	0.0420 (9)	−0.0058 (7)	0.0034 (7)	0.0005 (7)
O3	0.0541 (10)	0.0360 (8)	0.0530 (10)	−0.0022 (7)	0.0066 (8)	0.0032 (7)
O4	0.0497 (10)	0.0475 (10)	0.0544 (10)	−0.0141 (8)	0.0064 (8)	0.0109 (8)
N1	0.0462 (11)	0.0384 (10)	0.0442 (10)	−0.0019 (8)	−0.0114 (8)	−0.0042 (8)
C3	0.0378 (12)	0.0440 (13)	0.0563 (14)	−0.0015 (10)	−0.0132 (11)	0.0049 (11)
C4	0.0476 (14)	0.0594 (15)	0.0446 (13)	−0.0017 (12)	−0.0111 (11)	0.0145 (12)
C5	0.0461 (13)	0.0559 (15)	0.0391 (12)	0.0047 (11)	−0.0057 (10)	0.0017 (11)
C2	0.0523 (15)	0.0515 (14)	0.0471 (14)	0.0076 (11)	−0.0053 (11)	−0.0009 (11)
C1	0.0447 (13)	0.0499 (14)	0.0408 (12)	0.0022 (11)	−0.0042 (10)	0.0068 (10)
C6	0.0339 (11)	0.0451 (13)	0.0421 (12)	−0.0023 (9)	−0.0095 (9)	0.0018 (10)
C7	0.0347 (11)	0.0410 (12)	0.0429 (12)	−0.0040 (9)	−0.0074 (9)	0.0033 (10)
C8	0.0517 (14)	0.0466 (14)	0.0576 (15)	−0.0014 (11)	−0.0205 (12)	−0.0053 (11)
C9	0.0609 (16)	0.0458 (14)	0.0605 (16)	−0.0031 (12)	−0.0145 (13)	−0.0113 (12)
C10	0.0365 (11)	0.0355 (11)	0.0409 (12)	−0.0018 (9)	−0.0058 (9)	−0.0004 (9)
C11	0.0344 (11)	0.0356 (11)	0.0357 (11)	−0.0033 (9)	−0.0035 (9)	0.0012 (9)
C12	0.0387 (12)	0.0406 (12)	0.0353 (11)	−0.0027 (9)	−0.0023 (9)	0.0048 (9)
C13	0.0370 (12)	0.0391 (12)	0.0409 (12)	−0.0065 (9)	0.0035 (9)	0.0036 (9)
C17	0.088 (2)	0.0403 (15)	0.090 (2)	−0.0014 (14)	0.0106 (17)	0.0142 (14)
C18	0.088 (2)	0.0489 (17)	0.099 (2)	−0.0113 (15)	0.0167 (19)	0.0204 (16)
C14	0.0362 (12)	0.0484 (13)	0.0398 (12)	−0.0099 (10)	−0.0041 (9)	0.0014 (10)
C15	0.0344 (11)	0.0324 (11)	0.0374 (11)	−0.0025 (9)	−0.0022 (9)	−0.0037 (9)
C16	0.0356 (12)	0.0300 (11)	0.0389 (11)	−0.0037 (9)	−0.0019 (9)	0.0039 (9)
C23	0.0511 (14)	0.0352 (12)	0.0470 (13)	−0.0038 (10)	−0.0159 (11)	0.0054 (10)
C20	0.0424 (12)	0.0326 (11)	0.0353 (11)	−0.0032 (9)	−0.0049 (9)	0.0021 (9)
C25	0.0494 (14)	0.0416 (13)	0.0373 (12)	0.0056 (10)	−0.0010 (10)	−0.0001 (10)
C24	0.0655 (16)	0.0419 (13)	0.0341 (12)	0.0033 (11)	−0.0102 (11)	−0.0022 (10)
C19	0.0376 (12)	0.0375 (12)	0.0359 (11)	−0.0014 (9)	0.0014 (9)	−0.0016 (9)
C22	0.0406 (13)	0.0551 (15)	0.0444 (13)	−0.0027 (11)	−0.0019 (10)	0.0046 (11)
C21	0.0441 (13)	0.0570 (15)	0.0329 (11)	−0.0014 (11)	−0.0037 (10)	−0.0014 (10)
C26	0.0425 (13)	0.0394 (13)	0.0519 (14)	0.0008 (10)	−0.0051 (11)	−0.0094 (10)
C27	0.0523 (15)	0.0344 (12)	0.0560 (14)	0.0063 (10)	−0.0149 (12)	−0.0091 (10)
C28	0.0631 (18)	0.0515 (16)	0.081 (2)	0.0129 (13)	−0.0268 (15)	−0.0117 (14)
C29	0.091 (3)	0.0611 (19)	0.091 (2)	0.0178 (17)	−0.046 (2)	0.0050 (17)
C30	0.110 (3)	0.0517 (17)	0.072 (2)	0.0041 (17)	−0.037 (2)	0.0113 (14)
C31	0.085 (2)	0.0345 (13)	0.0558 (15)	−0.0017 (13)	−0.0175 (14)	0.0042 (11)
C32	0.0600 (15)	0.0276 (11)	0.0503 (13)	0.0005 (10)	−0.0154 (11)	−0.0041 (10)
C33	0.0483 (13)	0.0315 (11)	0.0467 (13)	−0.0038 (10)	−0.0060 (10)	−0.0002 (9)
C34	0.0519 (15)	0.0457 (14)	0.0708 (17)	−0.0119 (12)	0.0000 (13)	0.0096 (12)
C35	0.072 (2)	0.0567 (17)	0.083 (2)	−0.0164 (15)	0.0100 (16)	0.0156 (15)
C36	0.101 (2)	0.0453 (16)	0.0631 (18)	−0.0112 (16)	−0.0022 (17)	0.0142 (13)

### Geometric parameters (Å, º)

**Table:**

Cl1—C3	1.738 (2)	C17—H14A	0.9700
Cl2—C23	1.737 (2)	C17—H14B	0.9700
O1—C26	1.211 (3)	C18—H15A	0.9700
O2—C16	1.210 (2)	C18—H15B	0.9700
O3—C17	1.395 (3)	C14—C15	1.508 (3)
O3—C13	1.421 (3)	C14—H16A	0.9700
O4—C18	1.399 (3)	C14—H16B	0.9700
O4—C13	1.415 (2)	C15—C19	1.332 (3)
N1—C10	1.446 (3)	C15—C16	1.493 (3)
N1—C9	1.453 (3)	C23—C22	1.368 (3)
N1—C8	1.457 (3)	C23—C24	1.370 (3)
C3—C2	1.367 (3)	C20—C21	1.386 (3)
C3—C4	1.367 (3)	C20—C25	1.388 (3)
C4—C5	1.380 (3)	C20—C19	1.459 (3)
C4—H2	0.9300	C25—C24	1.377 (3)
C5—C6	1.386 (3)	C25—H21	0.9300
C5—H3	0.9300	C24—H22	0.9300
C2—C1	1.377 (3)	C19—H23	0.9300
C2—H4	0.9300	C22—C21	1.375 (3)
C1—C6	1.384 (3)	C22—H24	0.9300
C1—H5	0.9300	C21—H25	0.9300
C6—C7	1.515 (3)	C26—C27	1.469 (3)
C7—C8	1.529 (3)	C27—C28	1.370 (3)
C7—C11	1.556 (3)	C27—C32	1.388 (3)
C7—H7	0.9800	C28—C29	1.404 (4)
C8—H8A	0.9700	C28—H28	0.9300
C8—H8B	0.9700	C29—C30	1.350 (5)
C9—H9A	0.9600	C29—H29	0.9300
C9—H9B	0.9600	C30—C31	1.407 (4)
C9—H9C	0.9600	C30—H30	0.9300
C10—C33	1.529 (3)	C31—C36	1.388 (4)
C10—C26	1.562 (3)	C31—C32	1.404 (3)
C10—C11	1.592 (3)	C32—C33	1.406 (3)
C11—C16	1.527 (3)	C33—C34	1.358 (3)
C11—C12	1.547 (3)	C34—C35	1.422 (4)
C12—C13	1.510 (3)	C34—H34	0.9300
C12—H12A	0.9700	C35—C36	1.357 (4)
C12—H12B	0.9700	C35—H35	0.9300
C13—C14	1.512 (3)	C36—H36	0.9300
C17—C18	1.457 (4)		
			
C17—O3—C13	107.85 (18)	O4—C18—H15A	110.3
C18—O4—C13	108.50 (19)	C17—C18—H15A	110.3
C10—N1—C9	114.85 (18)	O4—C18—H15B	110.3
C10—N1—C8	108.72 (17)	C17—C18—H15B	110.3
C9—N1—C8	114.42 (19)	H15A—C18—H15B	108.6
C2—C3—C4	120.7 (2)	C15—C14—C13	107.91 (17)
C2—C3—Cl1	119.65 (19)	C15—C14—H16A	110.1
C4—C3—Cl1	119.61 (18)	C13—C14—H16A	110.1
C3—C4—C5	119.6 (2)	C15—C14—H16B	110.1
C3—C4—H2	120.2	C13—C14—H16B	110.1
C5—C4—H2	120.2	H16A—C14—H16B	108.4
C4—C5—C6	121.2 (2)	C19—C15—C16	117.20 (18)
C4—C5—H3	119.4	C19—C15—C14	126.80 (19)
C6—C5—H3	119.4	C16—C15—C14	115.89 (17)
C3—C2—C1	119.1 (2)	O2—C16—C15	121.13 (19)
C3—C2—H4	120.4	O2—C16—C11	121.43 (19)
C1—C2—H4	120.4	C15—C16—C11	117.25 (17)
C2—C1—C6	122.0 (2)	C22—C23—C24	121.6 (2)
C2—C1—H5	119.0	C22—C23—Cl2	118.41 (19)
C6—C1—H5	119.0	C24—C23—Cl2	119.95 (18)
C1—C6—C5	117.3 (2)	C21—C20—C25	117.6 (2)
C1—C6—C7	119.03 (19)	C21—C20—C19	122.78 (19)
C5—C6—C7	123.7 (2)	C25—C20—C19	119.6 (2)
C6—C7—C8	116.47 (19)	C24—C25—C20	121.6 (2)
C6—C7—C11	116.03 (17)	C24—C25—H21	119.2
C8—C7—C11	104.16 (18)	C20—C25—H21	119.2
C6—C7—H7	106.5	C23—C24—C25	118.6 (2)
C8—C7—H7	106.5	C23—C24—H22	120.7
C11—C7—H7	106.5	C25—C24—H22	120.7
N1—C8—C7	106.23 (18)	C15—C19—C20	128.7 (2)
N1—C8—H8A	110.5	C15—C19—H23	115.6
C7—C8—H8A	110.5	C20—C19—H23	115.6
N1—C8—H8B	110.5	C23—C22—C21	119.1 (2)
C7—C8—H8B	110.5	C23—C22—H24	120.5
H8A—C8—H8B	108.7	C21—C22—H24	120.5
N1—C9—H9A	109.5	C22—C21—C20	121.4 (2)
N1—C9—H9B	109.5	C22—C21—H25	119.3
H9A—C9—H9B	109.5	C20—C21—H25	119.3
N1—C9—H9C	109.5	O1—C26—C27	126.6 (2)
H9A—C9—H9C	109.5	O1—C26—C10	125.3 (2)
H9B—C9—H9C	109.5	C27—C26—C10	108.02 (19)
N1—C10—C33	117.84 (18)	C28—C27—C32	120.2 (2)
N1—C10—C26	111.85 (17)	C28—C27—C26	132.2 (3)
C33—C10—C26	101.27 (17)	C32—C27—C26	107.5 (2)
N1—C10—C11	100.67 (16)	C27—C28—C29	117.2 (3)
C33—C10—C11	112.28 (17)	C27—C28—H28	121.4
C26—C10—C11	113.53 (17)	C29—C28—H28	121.4
C16—C11—C12	111.26 (17)	C30—C29—C28	123.0 (3)
C16—C11—C7	112.58 (17)	C30—C29—H29	118.5
C12—C11—C7	112.80 (17)	C28—C29—H29	118.5
C16—C11—C10	107.85 (16)	C29—C30—C31	121.1 (3)
C12—C11—C10	112.76 (17)	C29—C30—H30	119.4
C7—C11—C10	98.95 (16)	C31—C30—H30	119.4
C13—C12—C11	112.03 (17)	C36—C31—C32	116.7 (3)
C13—C12—H12A	109.2	C36—C31—C30	127.7 (3)
C11—C12—H12A	109.2	C32—C31—C30	115.6 (3)
C13—C12—H12B	109.2	C27—C32—C31	122.9 (2)
C11—C12—H12B	109.2	C27—C32—C33	113.3 (2)
H12A—C12—H12B	107.9	C31—C32—C33	123.8 (2)
O4—C13—O3	106.47 (17)	C34—C33—C32	117.9 (2)
O4—C13—C12	109.99 (17)	C34—C33—C10	133.5 (2)
O3—C13—C12	110.72 (18)	C32—C33—C10	108.6 (2)
O4—C13—C14	111.57 (18)	C33—C34—C35	118.7 (3)
O3—C13—C14	107.20 (17)	C33—C34—H34	120.7
C12—C13—C14	110.77 (18)	C35—C34—H34	120.7
O3—C17—C18	105.4 (2)	C36—C35—C34	122.9 (3)
O3—C17—H14A	110.7	C36—C35—H35	118.6
C18—C17—H14A	110.7	C34—C35—H35	118.6
O3—C17—H14B	110.7	C35—C36—C31	119.9 (3)
C18—C17—H14B	110.7	C35—C36—H36	120.0
H14A—C17—H14B	108.8	C31—C36—H36	120.0
O4—C18—C17	106.9 (2)		
			
C2—C3—C4—C5	0.2 (4)	C14—C15—C16—C11	33.9 (3)
Cl1—C3—C4—C5	−178.79 (18)	C12—C11—C16—O2	142.6 (2)
C3—C4—C5—C6	−1.3 (4)	C7—C11—C16—O2	14.9 (3)
C4—C3—C2—C1	0.4 (4)	C10—C11—C16—O2	−93.2 (2)
Cl1—C3—C2—C1	179.47 (19)	C12—C11—C16—C15	−42.2 (2)
C3—C2—C1—C6	−0.1 (4)	C7—C11—C16—C15	−169.89 (17)
C2—C1—C6—C5	−0.9 (3)	C10—C11—C16—C15	82.0 (2)
C2—C1—C6—C7	178.8 (2)	C21—C20—C25—C24	1.0 (3)
C4—C5—C6—C1	1.6 (3)	C19—C20—C25—C24	178.7 (2)
C4—C5—C6—C7	−178.0 (2)	C22—C23—C24—C25	−0.2 (3)
C1—C6—C7—C8	146.4 (2)	Cl2—C23—C24—C25	−179.04 (17)
C5—C6—C7—C8	−34.0 (3)	C20—C25—C24—C23	−0.9 (3)
C1—C6—C7—C11	−90.4 (2)	C16—C15—C19—C20	176.2 (2)
C5—C6—C7—C11	89.2 (3)	C14—C15—C19—C20	−7.9 (4)
C10—N1—C8—C7	18.2 (2)	C21—C20—C19—C15	−33.0 (4)
C9—N1—C8—C7	148.1 (2)	C25—C20—C19—C15	149.5 (2)
C6—C7—C8—N1	141.39 (19)	C24—C23—C22—C21	1.1 (4)
C11—C7—C8—N1	12.3 (2)	Cl2—C23—C22—C21	179.93 (18)
C9—N1—C10—C33	−47.3 (3)	C23—C22—C21—C20	−0.9 (4)
C8—N1—C10—C33	82.3 (2)	C25—C20—C21—C22	−0.1 (3)
C9—N1—C10—C26	69.4 (2)	C19—C20—C21—C22	−177.7 (2)
C8—N1—C10—C26	−160.95 (18)	N1—C10—C26—O1	40.2 (3)
C9—N1—C10—C11	−169.71 (18)	C33—C10—C26—O1	166.5 (2)
C8—N1—C10—C11	−40.1 (2)	C11—C10—C26—O1	−72.9 (3)
C6—C7—C11—C16	82.6 (2)	N1—C10—C26—C27	−137.47 (19)
C8—C7—C11—C16	−148.03 (18)	C33—C10—C26—C27	−11.1 (2)
C6—C7—C11—C12	−44.4 (3)	C11—C10—C26—C27	109.4 (2)
C8—C7—C11—C12	85.0 (2)	O1—C26—C27—C28	7.9 (4)
C6—C7—C11—C10	−163.76 (18)	C10—C26—C27—C28	−174.5 (2)
C8—C7—C11—C10	−34.4 (2)	O1—C26—C27—C32	−169.1 (2)
N1—C10—C11—C16	162.25 (16)	C10—C26—C27—C32	8.5 (2)
C33—C10—C11—C16	36.0 (2)	C32—C27—C28—C29	−0.6 (4)
C26—C10—C11—C16	−78.1 (2)	C26—C27—C28—C29	−177.3 (3)
N1—C10—C11—C12	−74.5 (2)	C27—C28—C29—C30	1.2 (5)
C33—C10—C11—C12	159.28 (18)	C28—C29—C30—C31	−0.4 (5)
C26—C10—C11—C12	45.2 (2)	C29—C30—C31—C36	177.8 (3)
N1—C10—C11—C7	44.92 (18)	C29—C30—C31—C32	−0.9 (4)
C33—C10—C11—C7	−81.3 (2)	C28—C27—C32—C31	−0.8 (4)
C26—C10—C11—C7	164.59 (17)	C26—C27—C32—C31	176.7 (2)
C16—C11—C12—C13	−3.0 (2)	C28—C27—C32—C33	−179.3 (2)
C7—C11—C12—C13	124.57 (19)	C26—C27—C32—C33	−1.9 (3)
C10—C11—C12—C13	−124.37 (19)	C36—C31—C32—C27	−177.4 (2)
C18—O4—C13—O3	−7.6 (3)	C30—C31—C32—C27	1.5 (4)
C18—O4—C13—C12	112.5 (2)	C36—C31—C32—C33	1.1 (4)
C18—O4—C13—C14	−124.2 (2)	C30—C31—C32—C33	180.0 (2)
C17—O3—C13—O4	19.2 (3)	C27—C32—C33—C34	174.9 (2)
C17—O3—C13—C12	−100.4 (2)	C31—C32—C33—C34	−3.7 (4)
C17—O3—C13—C14	138.7 (2)	C27—C32—C33—C10	−5.6 (3)
C11—C12—C13—O4	−179.10 (17)	C31—C32—C33—C10	175.8 (2)
C11—C12—C13—O3	−61.7 (2)	N1—C10—C33—C34	−48.3 (4)
C11—C12—C13—C14	57.1 (2)	C26—C10—C33—C34	−170.6 (3)
C13—O3—C17—C18	−22.7 (3)	C11—C10—C33—C34	67.9 (3)
C13—O4—C18—C17	−6.3 (3)	N1—C10—C33—C32	132.3 (2)
O3—C17—C18—O4	17.9 (4)	C26—C10—C33—C32	10.0 (2)
O4—C13—C14—C15	171.50 (17)	C11—C10—C33—C32	−111.4 (2)
O3—C13—C14—C15	55.3 (2)	C32—C33—C34—C35	3.2 (4)
C12—C13—C14—C15	−65.6 (2)	C10—C33—C34—C35	−176.1 (2)
C13—C14—C15—C19	−156.5 (2)	C33—C34—C35—C36	−0.4 (4)
C13—C14—C15—C16	19.5 (3)	C34—C35—C36—C31	−2.2 (5)
C19—C15—C16—O2	25.5 (3)	C32—C31—C36—C35	1.9 (4)
C14—C15—C16—O2	−150.9 (2)	C30—C31—C36—C35	−176.9 (3)
C19—C15—C16—C11	−149.76 (19)		

### Hydrogen-bond geometry (Å, º)

**Table:**

*D*—H···*A*	*D*—H	H···*A*	*D*···*A*	*D*—H···*A*
C12—H12*A*···O1	0.97	2.27	3.066 (3)	139
C22—H24···O2^i^	0.93	2.35	3.172 (3)	148

Symmetry code: (i) *x*+1, *y*, *z*.
