# Supplementary figures and images for: Crystal structure of 5′′-(4-chloro­benzyl­idene)-4′-(4-chloro­phen­yl)-1′-methyltri­spiro[acenapthylene-1,2′-pyrrolidine-3′,1′′-cyclo­hexane-3′′,2′′′-[1,3]dioxane]-2(1H),6′′-dione
Source: Acta Crystallogr E Crystallogr Commun. 2015 Oct 3;71(Pt 11):o814–5. doi: 10.1107/S2056989015018034 (PMC4645075; doi:10.1107/S2056989015018034)

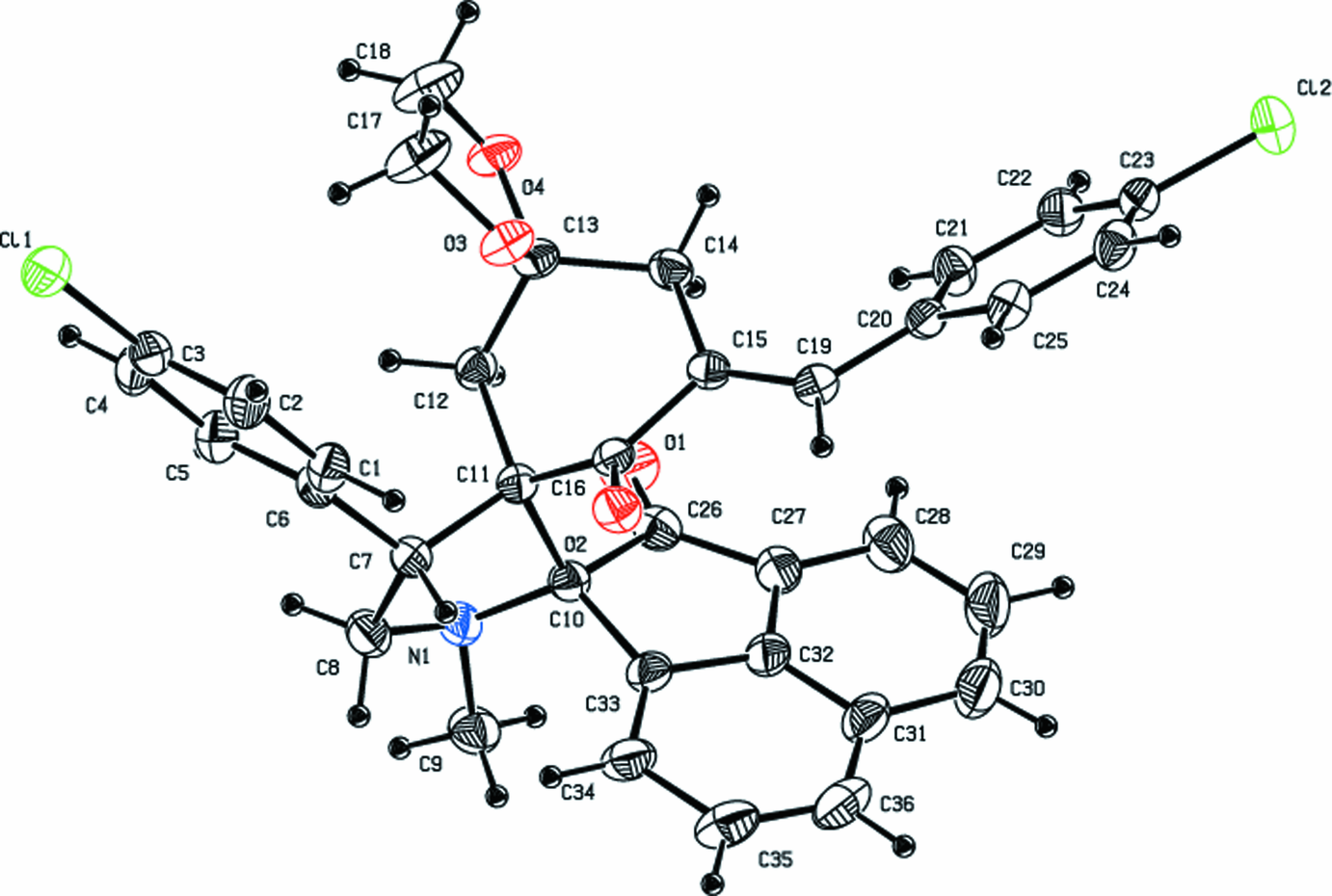

Supplement: Supplementary file 4 [file e-71-0o814-fig1.tif]

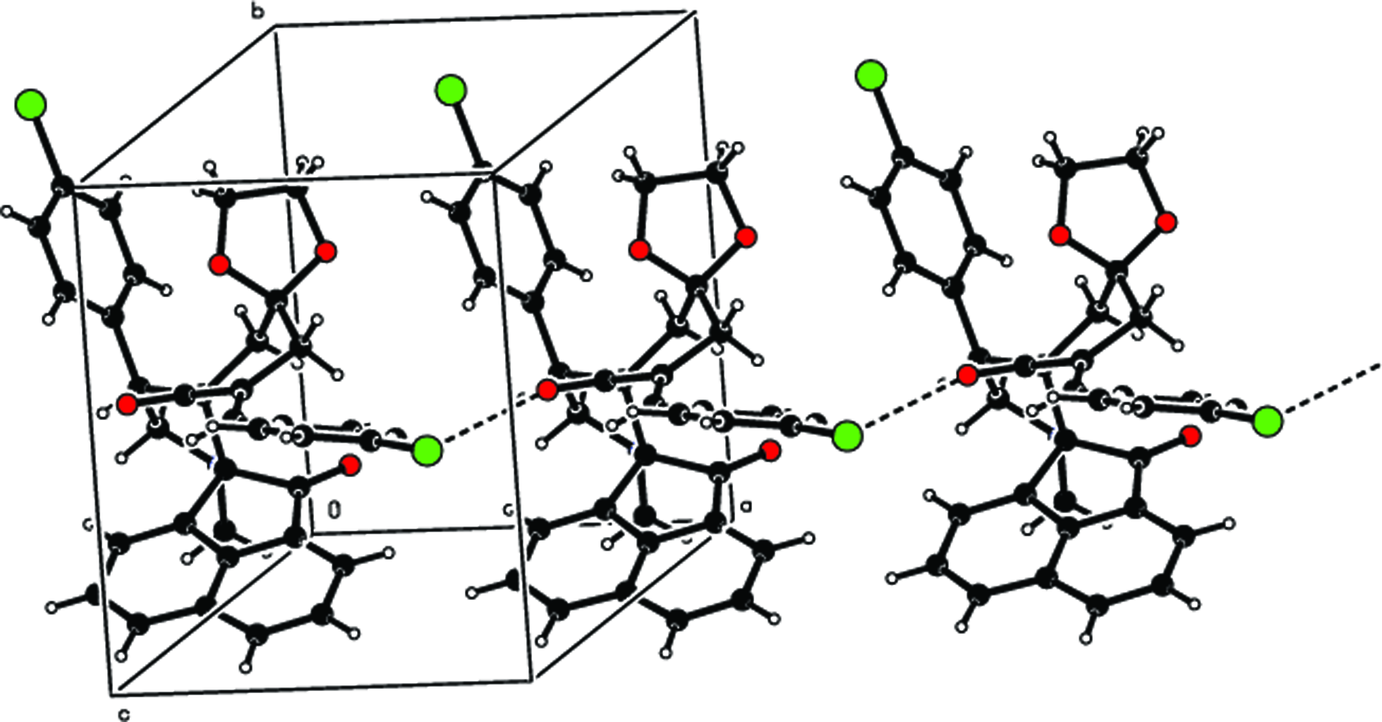

Supplement: Supplementary file 5 [file e-71-0o814-fig2.tif]
